# Gastric inflammatory pseudotumour secondary to *Actinomyces hordeovulneris* infection in a cat

**DOI:** 10.1186/s13620-016-0071-8

**Published:** 2016-08-26

**Authors:** Marco Pietra, Renato Giulio Zanoni, Angelo Peli, Barbara Brunetti, Nikolina Linta, Ombretta Capitani, Giuseppe Spinella

**Affiliations:** Department of Veterinary Medical Sciences, School of Agriculture and Veterinary Medicine, Alma Mater Studiorum - University of Bologna, Ozzano dell’Emilia (BO), 40064 Italy

**Keywords:** Cat, Gastritis, Pseudotumour, *Actinomyces hordeovulneris*

## Abstract

**Background:**

The authors report the first case of feline gastric actinomycosis associated with infection by *Actinomyces hordeovulneris*.

**Case presentation:**

A 4-year-old, neutered male, semi-feral European cat, with a 1 year history of chronic vomiting, was referred to the clinic. Abdominal ultrasound examination identified a hypoechoic focal transmural thickening with loss of normal wall layering and hyperechoic speckles at the gastric body. Initial gastroscopic examination showed a tumour-like gastric mass with an ulcerated depression at the level of the greater curvature. Histologic examination of endoscopic biopsy specimens was consistent with a severe lymphoplasmacytic gastritis. After 2 months, due to persistence of abdominal discomfort, surgical exploration and intraoperative sampling of gross abnormalities was recommended. Full thickness gastric wall biopsies, and fine needle aspiration of the gastric thickening and gastric lymph node, were performed. Histopathological examination identified a transmural pyogranulomatous gastritis. Aspirate samples of the gastric wall cultured positive, with colony morphology, biochemical testing and PCR of the 16 s rRNA gene compatible with *Actinomyces hordeovulneris*. After 4 months of treatment with cefovecin (8 mg/kg subcutaneously every 14 days), the vomiting completely resolved, as well as the ultrasonographic gastric alteration.

**Conclusion:**

This case report of feline gastric actinomycosis, caused by *Actinomyces hordeovulneris*, suggests that gastric bacterial infection should be considered in cases of focal gastric wall thickening associated with chronic vomiting in the cat, which may otherwise closely resemble neoplastic disease. Once a diagnosis of actinomycosis was obtained, a correct treatment with antibiotic therapy can resolve it.

## Background

Primary gastric actinomycosis is a rare gastric wall infection caused by *Actinomyces* species; it is rarely reported in people and dogs and is characterized by the formation of multiple gastric abscesses, draining sinuses with abundant granulation, and dense fibrous tissue [1]. To the authors’ knowledge, there have been no reports of gastric *Actinomyces* infection in cats, and the authors believe this condition is possibly under-diagnosed due to its resemblance to neoplastic disease. In this case report, the authors describe the unusual presentation of an intramural gastric infection with *Actinomyces hordeovulneris* in a cat.

Here we describe the clinical, sonographic, and endoscopic pattern that mimics a malignant tumour and describe the diagnostic procedures required in this case for diagnosis.

## Case presentation

A 4-year-old, 6-kg, neutered male, semi-feral European cat that had been regularly vaccinated (against feline viral rhinotracheitis, calicivirus, and panleukopenia), and dewormed was referred to the Bologna University Veterinary Teaching Hospital for a diagnostic evaluation to determine the cause of chronic vomiting. Vomiting had first been observed approximately 1 year previously, was initially intermittent, and had gradually increased without concurrent decrease in appetite or body weight. An abdominal ultrasound examination, performed by another veterinarian 6 months after the onset of clinical signs, had shown only a moderate enlargement of jejunal lymph nodes without modification of their echogenicity or shape, and without change of the echo structure of other abdominal organs. No laboratory analyses had been performed at that time. The owner reported a partial response to long-term treatment with ranitidine, which had been continued up until the time of referral. However, within the month prior to referral, vomiting had worsened to once daily and occurred most often in the morning, without association to feeding.

On clinical examination, the cat was bright and had a body condition score of 6/9. Rectal temperature was within reference interval (38.5 °C), mucous membranes were congested, and no modification in size or consistency of peripheral lymph nodes was noted. Respiratory and cardiovascular examinations were unremarkable. On abdominal palpation, an immobile, firm and painful mass, approximately 4 cm in diameter, was identified in the cranial abdomen.

Haematological and serum biochemical tests revealed only a mild leukopenia (white blood cell count 4.46 × 10^9^/L, reference interval 5.0–19.0 × 10^9^/L) associated with lymphocytopenia (lymphocyte count 0.31 × 10^9^/L, reference interval 1.5–7.0 × 10^9^/L). Results of faecal flotation were negative, and total thyroxine concentration was within reference interval (15 nmol/L, reference range 5–40 nmol/L). The cat was negative for serum feline leukaemia virus antigen^1^, but was positive for serum feline immunodeficiency virus antibodies^1^.

**Fig. 1:**
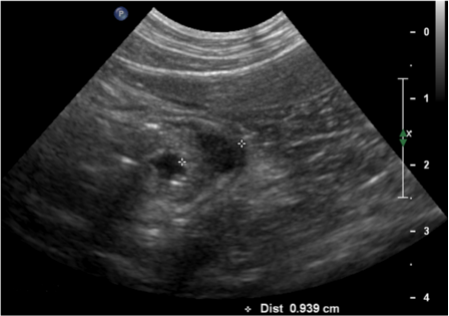
Initial abdominal ultrasound (cat). Transmural thickening (about 9 mm) of the gastric body wall with loss of normal wall layering

Abdominal ultrasound showed a hypoechoic transmural thickening (approximately 9 mm) at the gastric body, associated with loss of normal wall layering and hyperechoic speckles possibly consistent with focal fibrosis (Fig. 1). A moderate volume of anechoic fluid was seen in the gastric lumen. Gastric and portal lymph nodes were mildly enlarged and hypoechoic, but with normal shape. No other ultrasound findings were reported. Thoracic radiographs were unremarkable.

**Fig. 2:**
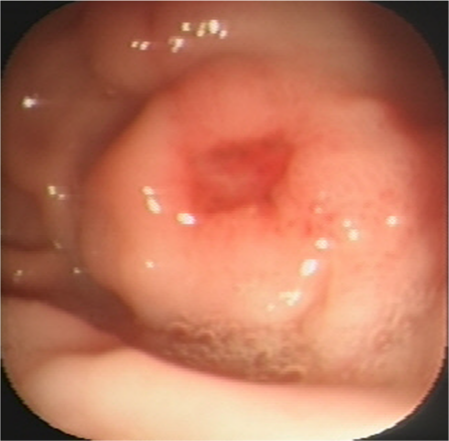
Initial endoscopy of the stomach (cat). Tumour-like gastric mass with an ulcerated depression at the level of the greater curvature between the fundus and gastric body

Endoscopic examination of the stomach showed a gastric mass with an ulcerated depression at the level of the greater curvature between the fundus and gastric body (Fig. 2). The endoscopic appearance of the duodenum was normal. Histological examination of biopsy specimens obtained from the base and edges of the region of ulceration was not compatible with a neoplastic process, and a healing ulcer associated with severe lymphoplasmacytic gastritis was diagnosed.

The cat was treated with marbofloxacin (5 mg/kg PO every 24 h for 10 day) and rabeprazole sodium (0.8 mg/kg PO every 24 h for 30 day) and fed a hypoallergenic diet. After initial clinical improvement, vomiting frequency subsequently increased gradually over the following 2 months to once daily, with a concurrent reduction of body weight (5.5 kg).

The cat was again presented, and examination identified abdominal discomfort, along with the palpable mass in the epigastric region that had been previously noted. Ultrasound examination identified similar changes to that seen previously with a focal hypoechoic wall thickening and loss of normal layering on the gastric greater curvature (increased from 9 to 11.8 mm from the previous ultrasound exam) extending for approximately 3 cm from the fundus to the gastric body; the mass was accompanied by marked enlargement of gastric (76 × 129 mm) and pancreatic-duodenal (57 × 97 mm) lymph nodes with an ovoid shape. At this presentation, given progression of disease and that previously findings may not have been representative, endoscopy together with celiotomy was recommended to allow direct observation of both sides of the gastric wall, and to allow surgical full thickness biopsy and aspiration of gastric lesion.

In addition an aspiration of the associated lymphadenopathy was advised.

**Fig. 3:**
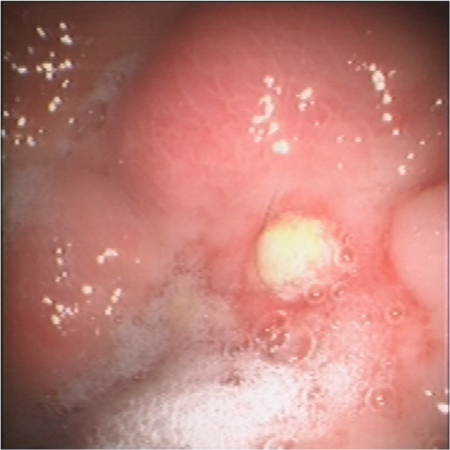
Second endoscopy of the stomach (cat). Performed 2 months after the initial endoscopy, shows the presence of a large whitish thickened mucosal area with a central part covered by fibrin at the level of the greater curvature in front of the pyloric antrum, indicating a healing phase ulcer

Endoscopic examination identified a large whitish, thickened mucosal area, the central region of which appeared to be covered by fibrin at the level of the greater curvature in front of the pyloric antrum, ascribable to pseudomembranous plaque, without evidence of an ulcer (Fig. 3). Based upon the appearance of the lesion, endoscopic biopsies were not performed.

At exploratory coeliotomy, evaluation of the gastric body identified a poorly delineated lesion at the greater curvature without macroscopic serosal alterations or adhesions with the omentum or other organs. Palpation of the gastric lesion confirmed a poorly delineated thickening of the gastric wall, with a tough and fibrous consistency. Enlarged gastric and pancreatic-duodenal lymph nodes were observed. Inspection of the other abdominal organs did not identify any gross pathological change. Two 14G full thickness core biopsies of gastric wall (stored in formalin for histologic procedures), and a fine needle aspiration of the gastric thickening (for a bacteriological examination) were performed. At biopsy, the consistency of the gastric wall appeared firm. A fine needle aspiration of the gastric lymph node (smeared on slides for a cytological examination) was obtained.

Histopathology of the gastric biopsy identified severe, chronic transmural pyogranulomatous gastritis. Cytological examination of the gastric lymph node indicated non-specific reactivity, with no evidence of neoplastic disease. On bacteriological examination, after 72 h of incubation in a capnophilic and anaerobic atmosphere, white colonies had grown on Columbia agar with 5 % sheep blood. The colonies had a molar tooth-like appearance and, after 7 days of incubation, showed slight haemolysis. The isolate was identified as *A. hordeovulneris* by chemical tests and 16S rRNA gene sequence, and based upon sensitivity testing was susceptible to penicillin, amoxicillin/clavulanate, cephalothin, cefovecin, tetracycline, enrofloxacin, and trimethoprim/sulfamethoxazole.

**Fig. 4:**
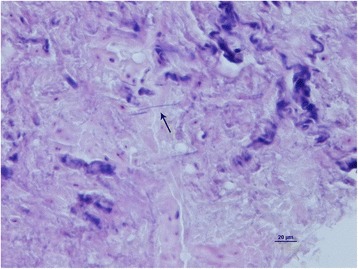
Gastric wall. Gram staining showing filamentous Gram-positive bacteria (*arrow*)

Following isolation of *A. hordeovulneris*, further staining of biopsy material was performed including Gram-staining and ZN-staining, in order to potentially identify organisms not previously observed in histopathologic sections. Gram-positive filamentous organisms were visible in multiple microscopic fields consistent with *Actinomyces spp.* (Fig. 4).

On the basis of these findings, treatment chosen was amoxicillin/clavulanate (12.5 mg/kg PO every 12 h for 4 months) and clebopride (5 μg/kg PO every 8 h for 2 months) with the purpose of increasing gastric empting. After 1 week, amoxicillin/clavulanate was replaced by cefovecin (8 mg/kg SC every 14 days for 4 months) due to a lack of cooperation of the patient in taking medication orally. The frequency of vomiting was reduced in the following week, and subsequently resolved completely.

On follow-up clinical examination, performed 4 months after surgery, 18 months after onset of clinical signs, body weight was increased again to 6.50 kg, and the only abnormal findings were mild abdominal tenderness and thickness of several intestinal loops. A complete blood count and biochemistry profile identified again a mild leukopenia (white blood cell count 4.04 × 10^9^/L, reference interval 5.0–19.0 × 10^9^/L) and lymphocytopenia (lymphocytes 0.27 × 10^9^/L, reference interval 1.5–7.0 × 10^9^/L).

**Fig 5:**
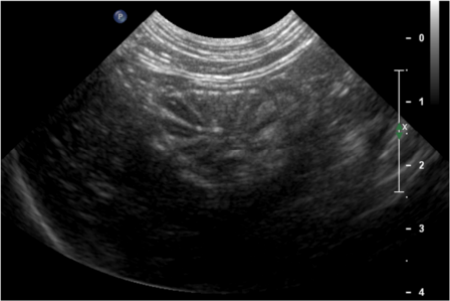
Third abdominal ultrasound (cat). Performed 4 months post-surgery, shows a complete healing of the gastric lesion with normal thickness and normal layering of the gastric wall

**Fig. 6:**
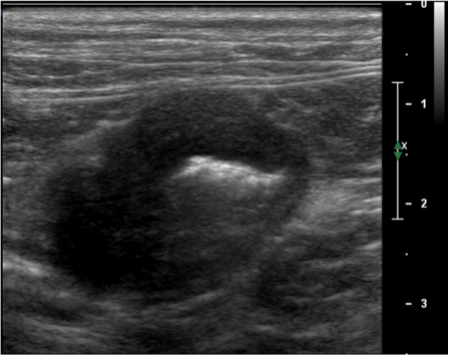
Third abdominal ultrasound (cat). Performed 4 months post-surgery, evidences a focal asymmetric hypoechoic thickening with loss of normal layering of the jejunal wall

Abdominal ultrasound identified complete resolution of the gastric lesion with normal thickness and almost normal layering of gastric wall (Fig. 5). The gastric lymph node was mildly enlarged (2.9 cm) and appeared hypoechoic with maintained shape. Unlike the previous ultrasound examinations, two focal asymmetric hypoechoic thickenings with loss of normal layering of the jejunal wall were observed. In one loop the wall measured 6.7 mm, for a length of approximately 3.2 cm (Fig. 6), and in the other the wall measured 3.1 mm, extending for a length of approximately 2 cm. A suggested surgical biopsy of jejunal wall thickening was declined by the owner.

Only ultrasound-guided aspirate of one of the intestinal lesions was performed and a cytological diagnosis of possible lymphoma was obtained. Screening thoracic radiography was therefore performed to evaluate extent and stage of disease, and was unremarkable.

Because it can be difficult in the cat to differentiate, by cytomorphology, lymphoma from inflammatory lymphoid cell infiltration of the gastrointestinal wall, a clonal T-cell receptor and B-cell receptor gene rearrangement analysis in feline lymphoid cells using GeneScan analysis was performed [2, 3]. In particular, formalin-fixed, paraffin-embedded gastric tissue (sample 1), stained smears of gastric lymph node cytological samples (sample 2) collected during the exploratory laparotomy, and stained smears of small intestinal aspirated on ultrasound and already diagnosed as suspected lymphoma (sample 3) were analysed. Polyclonal patterns for T-cell and B-cell receptor were identified in the samples 1 and 2, suggestive of reactive change, however a monoclonal pattern for T-cell receptor and polyclonal pattern for B-cell receptor were identified in sample 3, potentially compatible with a T-cell lymphoma.

Due to the semi-feral, aggressive nature of the cat, the owner declined multimodal chemotherapy [4], and chose treatment with methylprednisolone acetate injectable suspension (5 mg/kg SC every 30 day). At the time of the last telephone inquiry, 14 months after initial presentation (26 months after clinical sign onset), the owner reported that the cat remained free of clinical signs, showed no vomiting, and continued to have a good appetite and body condition.

This case report describes the diagnosis and treatment of a gastric pseudotumour due to *Actinomyces hordeovulneris*. The lesion is so called in human medicine because the chronic inflammatory process is able to induce granulomatous lesions mimicking a malignant tumour on clinical, ultrasonographic, and gross examination [5]. *Actinomyces* species are Gram-positive, branching, filamentous bacteria, and are common inhabitants of the feline oral cavity and gastrointestinal tract [6]. In cats *Actinomyces* have been associated with some cases of ocular [7], nasal [8], intracranial [9], pleural [10], peritoneal [11, 12], ileocolic junction [13] subcutaneous [14] and lymph nodal [15] disease.

To the authors’ knowledge, no report of focal gastric wall infection by *Actinomyces* has been previously described in the cat. Gastric and disseminated abdominal disease has been reported in a dog [16], and rare clinical cases of intramural gastric actinomycosis have been reported in people [1, 5, 17, 18]. Primary gastric actinomycosis is generally infrequent because the bacteria growth is inhibited by the acidic environment of the gastric lumen, and therefore the possibility of intramural translocation is unlikely [1, 18]. It is proposed in previous reports that gastric infection could be related to pharmaceutically-induced hypochlorhydria or to previous gastrectomy, both of which may subsequently increase gastric pH and thereby promote proliferation and opportunistic gastric wall infection [19]. In the case described herein, a possible reason for the development of gastric wall infection by *A. hordeovulneris* could be due to previous chronic therapy with ranitidine, a drug that is potentially capable to reduce hydrochloric acid secretion. Although ranitidine administered intravenously has been demonstrated to reduce gastric acidity in cats [20], administration of oral ranitidine may not result in similar effects [21].

*Actinomyces spp* infection has also been identified to occur following mucosal trauma, which may result from penetration via foreign material carrying the bacteria; this was not confirmed in our case but is a possibility.

Interestingly co-colonization of *A. hordeovulneris* with other bacteria was not observed in the case described, as has generally been observed in previous reports in people and animals [13] and is thought to enable an anaerobic environment appropriate for growth of actinomycetes. However, confirmation of *A. hordeovulneris* involvement in the lesion was late with respect to the onset of clinical signs, and followed administration of marbofloxacin, therefore co-colonization may have been missed.

We stress that marbofloxacin has to be considerate as a second-line antibiotic, and it should be limited to infections with identified susceptible bacteria. In the case presented here, marbofloxacin was administered following the first endoscopy due to the unclear history and chronic course of disease.

Common clinical signs reported both in canine and human gastric actinomycosis are chronic fever, emaciation, and vomiting [17]; also reported in humans is epigastric pain and gastrointestinal bleeding. Chronic vomiting and an irregular mobile mass in the mid-abdomen that was painful on palpation, without evident weight loss and fever, were the main clinical signs detected in the case reported here, similar to those reported by Sharman et al. [13]. Persistent leukopenia was identified in the current case, in comparison to the finding of leucocytosis, hyperglobulinaemia and hypoalbuminaemia reported by Sharman et al. [13] which are more typical findings of chronic inflammatory disease. This condition could be explained, in our case, by co-infection with the retrovirus FIV, which is able to sometimes induce constant leukopenia, as already reported by Shelton et al. [22]. Equally, for the current case leucopenia could reflect consumption due to the presence of a gastric granuloma, although given its persisteance despite clearance of the actinomycetoma.

Histopathology is required to differentiate actinomycetomas from neoplastic disease, given that clinical, imaging and endoscopic findings are non-specific and mimick that of neoplasia.

In previous studies, gastric actinomycosis was diagnosed directly on histological analysis of a resected specimen by evidence of colony gram-positive filamentous rods with a surrounding layer of neutrophils (macroscopically so-called “sulphur granules”) [1, 13, 16, 17, 19]. In our case, sulphur granules were not present, and histopathologic examination did not allow identification of bacterial colonization within the pyogranulomatous inflammation using standard staining techniques. Involvement of filamentous bacteria was only confirmed following retrospective gram-staining once positive culture results were obtained. This finding leads the Authors to suggest that staining techniques directed towards identifying infectious agents should be performed where similar conditions are suspected, for example where pyogranulomatous inflammation is detected.

Even so, in our case, an aetiologic diagnosis also required bacterial culture of the gastric layer aspirate, followed by identification by PCR of the organism isolated.

The therapeutic approach of treating gastric actinomycosis in humans is controversial because while some authors [1] recommend surgical resection of the involved gastric tissue followed by administration of antibiotics (ampicillin, cephalosporin, tetracycline, macrolide or clindamycin) for a prolonged period (up to 12 months) with a favourable outcome, others indicate exclusive treatment with antibiotics without surgical debridement [16]. In the present case report, we observed complete restoration of the original condition of the gastric layer with 4 months’ treatment with antibiotics.

It should also be noted that concurrent FIV infection could be a factor that contributed to both gastric infection by *A. hordeovulneris* and the development of the suspected intestinal lymphoma.

The authors would note, that the differentiation of intestinal lymphoma from gastrointestinal inflammation should follow a stepwise diagnostic algorithm that first uses histologic assessment, followed by immunophenotyping and then PCR to determine clonality of lymphocytes [23]. In our case it was not possible to follow the correct diagnostic algorithm as the owner declined intestinal biopsy, allowing only aspiration for cytology. Therefore only cytology together with assessment for clonal T- and B-cell receptor rearrangement contributed to the diagnosis of suspected lymphoma. As the cell population harvested by not be entirely representative using this methodology, the results of clonality analysis might also not be representative in this case. In addition one known pitfall of clonality analysis is that chronic antigenic stimulation, such as may occur with IBD, can also result in an expanded clonal population of lymphocytes and false positive results. The diagnosis of lymphoma reached here should therefore be interpreted with caution, especially given the long-term response to minimal therapy.

## Conclusions

Gastric actinomycosis remains a diagnostic challenge due to the rarity of the infection and the similarity of lesions with neoplastic disease. In any case, especially in cats with predisposing factors such as conditions able to induce immunodeficiency, clinicians should suggest the execution of bacterial isolation from the gastric wall for a definitive diagnosis.

## Footnotes

1 Snap® FIV/FeLV Combo Plus, IDEXX Laboratories, Milano (Italy).
